# Notes and News

**Published:** 1904-03-12

**Authors:** 


### Notes and News.

The King has been pleased to sanction several promotions in and appointments to the Order of the Hospital of St. John of Jerusalem in England. Colonel (Hon. Surgeon-General) William Duncan Campbell Williams, C.B., from Hon. Associate, Dr. George Thomson, from Hon. Associate, and Lord Northcote, become Knights of Grace; Lady Northcote, a Lady of Grace, and Dr. William Allan Jamieson Esquire.

---

Sir Charles Wyndham has consented to preside at the festival dinner at the Hôtel Cecil in connection with the centenary of the Royal London Ophthalmic Hospital, City Road, on Friday, April 22.

---

As it has been found impracticable to hold the Royal Waterloo Hospital festival dinner on the Saturday in Ascot week, it will take place on the following Monday, June 20, in the new buildings of the Savoy Hotel.

---

A concert, under the patronage of the Duchess of Connaught and the Duchess of Fife will be given at the Queen's Hall, Langham Place, on March 23rd, in aid of the North-Eastern Hospital for Children, Hackney Road, with the object of raising £5,000 to enable the hospital to carry on its largely increased work during the present year free from debt.

---

In addition to the donation from the King to the fund for the Institute of Medical Sciences, projected by the University of London, Mr. Alfred Beit has contributed £5,000, Mr. H. T. Butlin £1,000, Messrs. Rothschild and Son, £500, Dr. J. K. Fowler £250, and Sir William Church, Bart., Mr. John Tweedy, Sir Henry Roscoe, and Sir E. Cooper Perry, M.D., £100 each.

---

It is announced that the Craggs Research Prize for the ensuing year will be awarded to the past or present student of the London School of Tropical Medicine who makes the most valuable contribution to Tropical Medicine. Essays of candidates must be sent in to the medical tutor at the London School of Tropical Medicine, on or before October 1. The value of the prize is £50.

---

The Bishop of London will preach in aid of the rebuilding fund of St. Bartholomew's Hospital in St. Paul's Cathedral on Sunday morning, July 24.

---

The Ecclesiastical Commissioners have intimated to the Principal of King's College that they are prepared to contribute £2,500 to the King's College Hospital Removal Fund.

---

The Hospital for Epilepsy and Paralysis, Maida Vale, has benefited to the extent of £236 by the proceeds of the *café chantant* organised at the Royal Society of British Artists on February 25.

---

The Grosvenor Hospital for Women and Children, Vincent Square, Westminster, has benefited to the extent of £408 by the ball organised by Lady Aberdare at the Whitehall Rooms on February 15th.

---

Lord Knollys has informed Sir Trevor Lawrence that the King hopes that it may be in his power to lay the first stone of the new St. Bartholomew's Hospital, and that he will probably be accompanied by the Queen. The most convenient time to the King, Lord Knollys adds, would be a day about the end of June or at the beginning of July.

---

At a meeting on Tuesday of members of the House of Commons connected with the medical profession, it was agreed to form a committee to arrange for concerted action in the House on matters affecting public health. Dr. Farquharson presided, and there were present Sir Michael Foster, Sir J. Batty Tuke, Dr. Hutchinson, and Dr. Rutherfoord Harris. Sir Michael Foster has agreed to act as Hon. Secretary to the Committee.

---

Dr. Alexander Davidson, Emeritus Professor of Pathology in University College, Liverpool, which he helped to establish, died last week at the age of 67. He was admitted a Member of the Royal College of Physicians in 1874 and a Fellow in 1885. In conjunction with his colleague and schoolfellow, Sir William Mitchell Banks, Dr. Davidson was instrumental in building the new Royal Infirmary at Liverpool, to which he was for long senior physician.

---

It was intimated on Saturday by the War Office that the services of eight dental surgeons are authorised for duty with troops in the United Kingdom from April 1 next. They will be required to devote their whole time to Army duty, and will receive an inclusive salary of £365 per annum and travelling expenses. The period of engagement will be for one year only, and under conditions specified in the contract. The necessary dental appliances will be provided. It is probable that the dental surgeons will be stationed at Aldershot, Devonport, Cork, Edinburgh, Portsmouth, Dublin, Colchester, and Woolwich; but they will be expected to give attendance as directed by general officers commanding at all stations in the commands mentioned. Applications should be addressed to the Secretary of the Army Council, 68 Victoria Street, S.W., not later than Saturday, March 12, and should be accompanied by evidence of age and qualifications, and not more than three recent testimonials.

